# Elevated leukotriene B4 and 8-isoprostane in exhaled breath condensate from preterm-born infants

**DOI:** 10.1186/s12887-023-04210-y

**Published:** 2023-08-05

**Authors:** Rhea Urs, Rubi Ni Chin, Naomi Hemy, Andrew C. Wilson, J. Jane Pillow, Graham L. Hall, Shannon J. Simpson

**Affiliations:** 1 School of Allied Health, Curtin University, Perth, WA Australia; 2 Wal-yan Respiratory Centre, Telethon Kids Institute, Perth, WA Australia; 3 Perth Children’s Hospital, Perth, WA Australia; 4 School of Human Sciences, University of Western Australia, Perth, WA Australia

**Keywords:** Bronchopulmonary dysplasia, Preterm, Infant, Inflammation

## Abstract

**Background:**

Inflammation and oxidative stress play a key role in the development of bronchopulmonary dysplasia (BPD), possibly contributing to persistent respiratory morbidity after preterm birth. We aimed to assess if inflammatory markers were elevated in exhaled breath condensate (EBC) of infants born very prematurely (< 32 weeks gestation) at 12–16 corrected months of age, and if increased levels were associated with BPD diagnosis and respiratory morbidity.

**Methods:**

EBC samples and respiratory questionnaires were collected from 15 term-born infants and 33 preterm-born infants, 12 with a neonatal BPD diagnosis. EBC samples were analysed for leukotriene B4 (inflammation) and 8-isoprostane (oxidative stress) concentrations using enzyme-linked immune-assays. Differences between groups were analysed by Kruskal-Wallis Test with post-hoc comparisons, independent samples t-test or Mann-Whitney U test depending on normality of the data.

**Results:**

Leukotriene B4 and 8-isoprostane levels were elevated in exhaled breath condensate of preterm-born infants compared to those born at term (mean difference [95% CI]; 1.52 [0.45, 2.59], p = 0.02; 0.77 [0.52, 1.02], p < 0.001, respectively). Leukotriene B4 and 8-isoprostane levels were independent of BPD diagnosis and respiratory morbidity over the first year of life.

**Conclusions:**

Infants born very prematurely exhibit elevated markers of airway neutrophilic inflammation and oxidative stress beyond the first year of life, regardless of a neonatal diagnosis of chronic lung disease or respiratory morbidity during infancy. These findings may have implications for future lung health.

**Trial Registration:**

N/A.

**Supplementary Information:**

The online version contains supplementary material available at 10.1186/s12887-023-04210-y.

## Background

Infants born very prematurely (< 32 weeks gestation) are often exposed to pro-inflammatory stimuli prior to and following preterm birth. These pro-inflammatory stimuli include chorioamnionitis, respiratory and systemic infections and lung injury from prolonged mechanical ventilation or oxygen supplementation. Inflammation and oxidative stress play a key role in the development of the chronic lung disease of prematurity, known as bronchopulmonary dysplasia (BPD) [1–3]. Increased pro-inflammatory cytokines, chemokines, neutrophils and reactive oxygen species are observed in the tracheal aspirates and bronchoalveolar lavage of neonates who go on to develop BPD and are associated with decreased pulmonary vascularisation and more simplified alveoli [1, 3]. Early life inflammatory events may predispose those born very prematurely to persistent respiratory morbidity throughout childhood, given recently described associations between perinatal inflammation and airway obstruction at 12 years old in those born preterm [4].

Several small studies suggest that pulmonary inflammation may persist through childhood [5–7]. Sputum samples obtained from children with BPD, born in the pre-surfactant era, indicate a 16-fold increase in sputum neutrophils and a 3-fold increase in sputum interleukin-8 [5]. Systemically, increased urinary leukotriene E4 [6] has been detected in school-aged children born preterm compared to those born at term. Finally, Filippone et al. measured increased 8-isoprostane levels, a marker of oxidative stress, in the exhaled breath condensate (EBC) of preterm-born adolescents both with and without a neonatal diagnosis of BPD in comparison to term-born controls [7].

We recently published a new, modified, method to collect exhaled breath condensate in infants [8]. EBC is a bio-fluid specific to the airways. Collection of EBC is non-invasive and relatively easy, which is particularly useful in an infant population [9]. Of particular interest, our modified EBC collection method may allow the identification of airway inflammation during infancy in survivors of preterm birth to detect those who may be at risk of ongoing respiratory morbidity throughout childhood.

This study aimed to assess if markers of inflammation and oxidative stress were elevated in the EBC of preterm-born infants compared to those born at term, and to determine if these markers are associated with poorer lung health in the first year of life. It was hypothesised that inflammatory and oxidative stress markers will be increased in EBC collected from preterm-born infants compared to those born at term. It was also hypothesised that increased inflammatory marker levels at 12–16 months are associated with a neonatal diagnosis of BPD, increased neonatal respiratory support and increased respiratory symptoms in the first year of life.

## Methods

### Participants

Preterm-born infants between 12 and 16 months corrected postnatal age (cPNA) attended Princess Margaret (now known as Perth Children’s) Hospital as part of the Preterm Infant Functional and Clinical Outcome (PIFCO) follow-up study (ACTRN12613001062718) [10], see Supplementary Fig. 1 (Additional File 1). All preterm-born participants were born at 32 weeks gestation or less between September 2013 and February 2017 and admitted to King Edward Memorial Hospital (KEMH) in Perth, Western Australia. Participants born preterm were classified as having bronchopulmonary dysplasia if they received 28 days of oxygen supplementation or more, as assessed at 36 weeks postmenstrual age [11]. At their study appointment, preterm-born participants underwent infant lung function testing, including EBC collection, performed under sedation with 80 mg/kg oral chloral hydrate [12]. A modified version of the International Study of Asthma and Allergies in Childhood (ISAAC) questionnaire [13] and general health questionnaire was completed by the infant’s parents which asked about the infant’s birth information, feeding, diet and respiratory health during the first year of life including cough, wheeze, hay fever, infections, medications and hospital admissions. The questionnaire also requested information on the infant’s general health, family history and exposure to respiratory irritants. Medical history was obtained from hospital records.

Term-born healthy infants aged between 9 and 18 months were recruited from the community and their study participation involved a home visit. At the home visit, a general health questionnaire was completed by the infant’s parents. EBC was collected during natural sleep during their regular sleep time. Term-born participants were born at or after 37 weeks gestation with no history of wheeze and/or recurrent cough, doctor diagnosis of respiratory disease or any neonatal respiratory disease.

Both term- and preterm- born infants were excluded from the study if they had major congenital abnormalities influencing cardiorespiratory function.

### Ethics

Written informed consent was received from the parents/guardians of the participants. The PIFCO follow-up study was approved by the Child and Adolescent Health Service (CAHS) HREC [Approval #2014083EP]. Sample analysis was approved by Curtin University (HRE2020-0097). Approval for the recruitment and sample collection from healthy term-born infants was obtained from Curtin University (HRE2018-0407).

### EBC Collection

EBC was collected using an R-Tube collection device (Respiratory Research Inc, Charlottesville, VA) adapted for collection in neonates and infants by reducing dead-space, as previously described by our group [8]. EBC was collected with the infants in supine position during tidal breathing. Infants breathed into the device through an infant face-mask (size 1, Laerdal Medical AS, Stavanger, Norway) placed over the infant’s nose and mouth to create a leak-free seal. Collection took place over 10–15 min of tidal breathing through the R-Tube [8]. After collection was complete, the condensate was aliquoted into Eppendorf tubes and stored at -80 degrees Celsius [8].

### EBC Analysis

Samples were analysed for leukotriene B4 and 8-isoprostane concentrations using enzyme-linked immune-assays (ELISA). Any samples with insufficient volume for both assays were prioritised for 8-isoprostane analysis. ELISA buffer, ELISA standards, samples, AChE tracers and ELISA antiserums for leukotriene B4 and 8-isoprostane were added to the assay plates according to the protocol outlined by the Cayman Chemical Leukotriene B4 EIA Kit (Item No. 520,111) and 8-Isoprostane EIA Kit (Item No. 516,351). A standard curve was plotted using the absorbance readings of the maximum binding, non-specific binding and standard wells. The standard curve was then used to determine the concentrations of leukotriene B4 and 8-isoprostane in the EBC samples. Measures below the limit of detection were assigned a concentration of 0 pg/mL.

### Statistical analysis

Normally distributed data are presented as means and standard deviations. Non-normally distributed data are presented as medians and interquartile ranges. To assess differences in demographic and respiratory history data between the term and preterm group, independent samples t-test or Mann-Whitney U test were used depending on normality of the data. Similarly, a sub-analysis was conducted within the preterm group between those with and without BPD. Mann-Whitney U test was used to determine if the concentration of biomarkers was elevated in those born preterm, compared to term. A sub-analysis was conducted using the Kruskal-Wallis Test to examine the differences between the term, preterm with BPD and without BPD groups, with Bonferroni correction for multiple tests. Bivariate correlation (Spearman’s rho) was used to assess associations between biomarker levels and length of neonatal respiratory support, and Mann-Whitney U Tests to determine whether biomarker levels were elevated in the presence/absence of symptoms, hospitalisations, medication usage and respiratory diagnosis. Analysis was performed using SPSS for Windows [14]. A p-value of < 0.05 was considered significant for all analyses. Power analysis using G*Power [15] found that 42 participants were required to achieve a power > 80% to detect whether markers of inflammation were elevated in those born preterm, assuming a large effect size using Cohen’s criteria (0.8) and significance criterion of α = 0.05.

## Results

Exhaled breath condensate samples were collected from 15 term-born infants (mean age 14.4 ± 2.9 months) and 33 preterm-born infants (mean age 14.4 ± 1.0 months), 12 of whom had a neonatal diagnosis of BPD. Of those with BPD, 7 were classified as moderate/severe BPD. All infant EBC samples were analysed for 8-isoprostane, with enough sample for leukotriene B4 analysis for 9 (60%) term-born participants and 29 (87.9%) preterm-born participants, 9 (75%) with BPD. 3 term infant samples had levels below the limit of detection (2 for leukotriene B4, 1 for 8-isoprostane), these values were imputed as zero. Anthropometric, birth and respiratory history information for infant participants are found in Table 1.

**Table 1:** Demographic, anthropometric and respiratory history information for term and preterm-born infants

	Term Controls	Preterm Infants	Preterm, no BPD	Preterm with BPD
Participants, n	15	33	21	12
Corrected age (months)	14.4 ± 2.9	14.4 ± 1.0	14.2 ± 1.0	14.7 ± 0.9
Male, n (%)	9 (60)	25 (75.7)	18 (85.7)	7 (58.3)
Weight (kg)	9.8 ± 1.4	9.9 ± 1.4	9.8 ± 1.0	10.2 ± 1.9
Gestational age at birth (w)	39 ± 1.2	27.7 ± 2.2*	29.0 ± 1.4	25.5 ± 1.3#
Birth weight (kg)	3.2 (3.0–3.5)	0.96 (0.78–1.21)*	1.13 (0.92–1.36)	0.82 (0.71–0.92)#
Birth weight centiles	56.8 (36.7–83.1)	55.0 (26.0–72.0)	40.0 (19.0–66.0)	68.0 (57.3–85.3) #
Chorioamnionitis, n (%)	n/a	15 (45.5)	6 (28.6)	9 (75)#
Days of ventilation	n/a	0.53 (0.29–3.83)	0.38 (0-0.58)	13.6 (2.55–42.5) #
Days of CPAP	n/a	31.8 (6.25–49.8)	10.3 (5.42–30.3)	51.5 (47.2–56.6) #
Days of oxygen	n/a	4.17 (0.54–68.1)	0.75 (0.25–2.54)	90.4 (64.3-125.6) #
Wheeze ever, n (%)	n/a	19 (57.6)	12 (57.1)	7 (58.3)
Asthma medication use ever, n (%)	n/a	6 (18.2)	2 (9.5)	4 (33.3)
Respiratory diagnosis ever (bronchiolitis, bronchitis, croup, whooping cough or pneumonia), n (%)	n/a	17 (51.5)	11 (52.4)	6 (50.0)
Respiratory hospitalisation ever (post initial discharge from NICU), n (%)	n/a	11 (33.3)	7 (33.3)	4 (33.3)
EBC Leukotriene B_4_ (pg/mL)	0.3 (0.0-1.4)	1.9 (1.2–3.3)*	1.9 (1.1–3.2)	2.3 (1.5–2.7)
EBC 8-isoprostane (pg/mL)	0.2 (0.0-0.3)	0.9 (0.6–1.2)**	0.9 (0.6–1.3)	0.9 (0.7-1.0)

Data expressed as mean ± SD, median (IQR) or number (percentage)

*significantly different between term and preterm-born groups (p < 0.05)

**significantly different between term and preterm-born groups (p < 0.01)

# significantly different between preterm infants with and without BPD (p < 0.05)

**Fig. 1:**
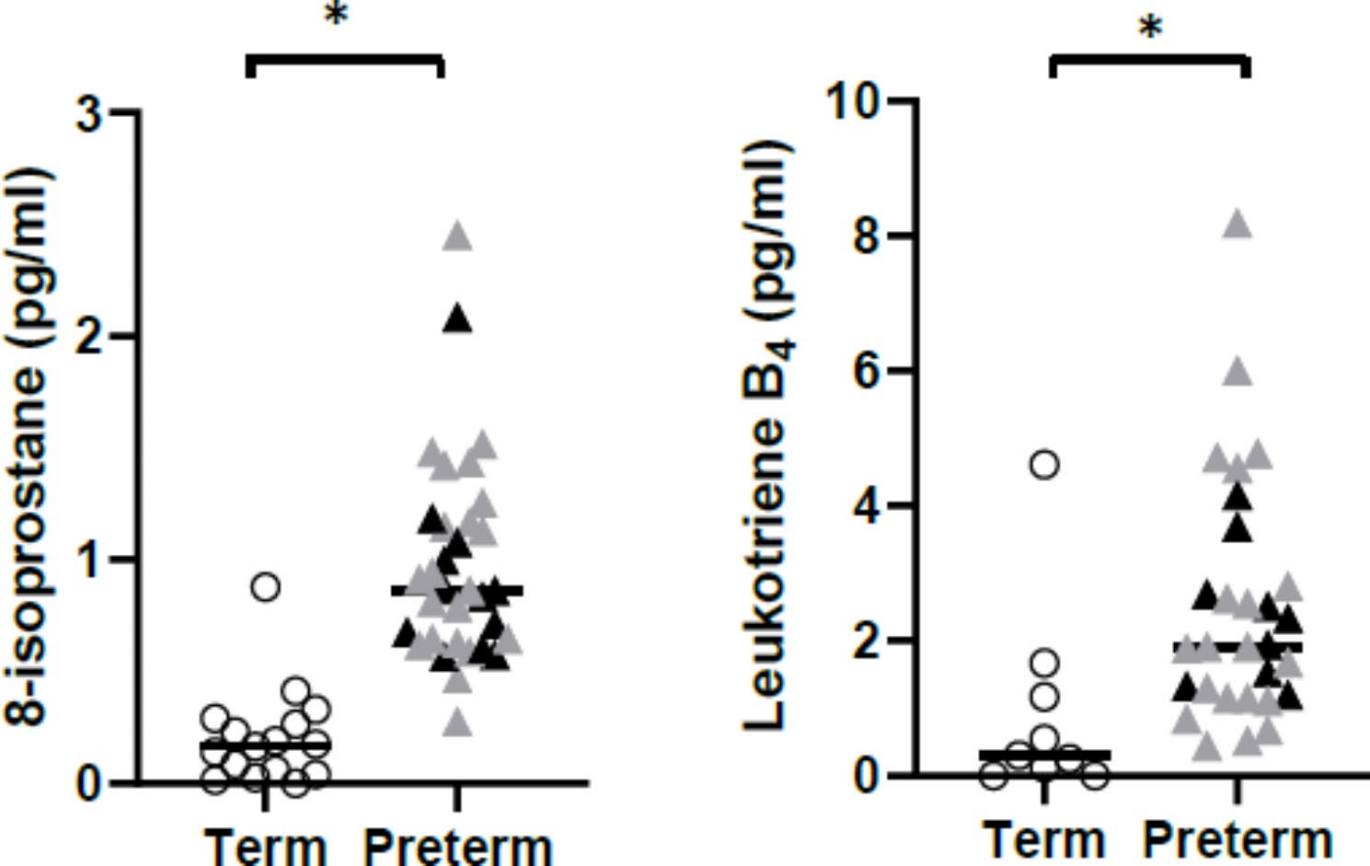
8-isoprostane and leukotriene B4 levels detected in the exhaled breath condensate of term and preterm-born infants. Open circles denote those born at term. Grey triangles denote infants born preterm without a neonatal diagnosis of BPD and black triangles denote infants with a neonatal diagnosis of BPD. The horizontal bar corresponds to the median value of each group. *significantly different between term and preterm-born groups (p < 0.05)

Leukotriene B4 and 8-isoprostane levels were elevated in the exhaled breath condensate of preterm-born infants compared to those born at term (mean difference [95% CI]; 1.52 [0.45, 2.59], p = 0.02; 0.77 [0.52, 1.02], p < 0.001, respectively) (Fig. 1). Kruskal-Wallis Testing showed that levels of leukotriene-B4 and 8-isoprostane were elevated in both preterm infants with and without BPD compared to term, however, no difference between those born preterm with and without BPD was found (p = 0.59 for leukotriene B4; p = 0.64 for 8-isoprostane;). There were also no associations found between biomarker levels and those whose mother did or did not have chorioamnionitis, length of gestation, mechanical ventilation, continuous positive airway pressure (CPAP) or oxygen supplementation (Table 2).

**Table 2:** Association between gestational age, indicators of postnatal respiratory support and EBC markers of inflammation and oxidative stress in very preterm infants

	Leukotriene B4 (pg/mL)			8-isoprostane (pg/mL)		
	Spearman’s rho	Sig. (2-tailed)	N	Spearman’s rho	Sig. (2-tailed)	N
Gestational age (weeks)	− 0.198	0.303	29	0.157	0.382	33
Duration of ventilation (hours)	0.123	0.524	29	− 0.335	0.056	33
Duration of CPAP (hours)	0.183	0.342	29	− 0.015	0.932	33
Duration of HHF (hours)	0.211	0.273	29	− 0.257	0.149	33
Duration of supplemental oxygen (hours)	0.116	0.573	26	0.028	0.882	30

**Fig. 2:**
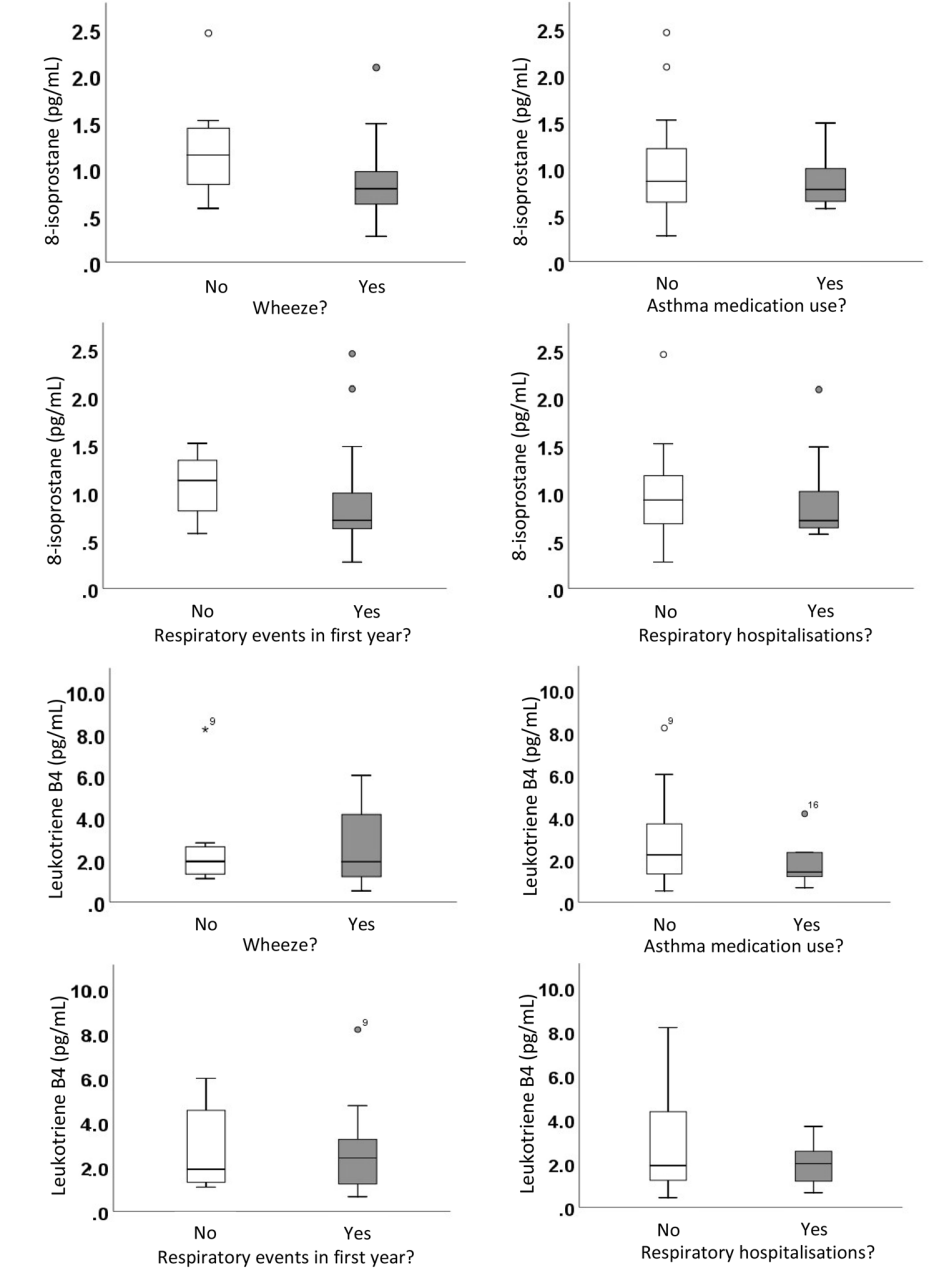
8-isoprostane and leukotriene B4 levels in exhaled breath condensate from preterm-born infants aged 12–15 months who did and did not report a history of wheeze, asthma medication use, respiratory events in the first year of life (either bronchiolitis, bronchitis, croup or pneumonia) or respiratory hospitalisation. There was no significant difference (p > 0.05) in 8-isoprostane or leukotriene B4 levels between those who did and did not report a history of respiratory symptoms, medication use or hospitalisations

Additionally, leukotriene-B4 and 8-isoprostane levels were not different in preterm infants who did and did not report a history of wheeze (mean difference [95% CI]; -0.01 [-1.66, 1.63], p = 0.99; 0.31 [-0.11, 0.73], p = 0.13, respectively), asthma medication use (mean difference [95% CI]; 0.89 [-0.53, 2.30], p = 0.20; 0.14 [-0.24, 0.52], p = 0.44, respectively), any respiratory events (such as upper respiratory tract infections (URTI), bronchitis, bronchiolitis, croup, pneumonia) (mean difference [95% C]; 0.03 [-1.50, 1.55], p = 0.97; 0.10 [-0.25, 0.45], p = 0.56, respectively) or hospitalisations for respiratory reasons (mean difference [95% CI]; 0.84 [-0.32, 2.00], p = 0.15; 0.07 [-0.29, 0.44], p = 0.68, respectively) (Fig. 2).

## Discussion

This study shows that very preterm-born infants at 12–16 months corrected postnatal age (cPNA) have persistent airway inflammation evidenced by elevated leukotriene B4 and 8-isoprostane in EBC of the preterm infants compared to those born at term. No associations were found between biomarker levels and neonatal diagnosis of BPD, increased neonatal respiratory support or increased respiratory symptoms in the first year of life.

The increased levels of leukotriene B4, a marker of neutrophilic inflammation [16], and 8-isoprostane, a marker of oxidative stress [17], in EBC from preterm-born infants may be indicative of persisting lung injury from premature birth, subsequent intervention [18] or other insults during the first year of life. Leukotriene B4 is a potent neutrophil chemoattractant [16], while 8-isoprostane is a mediator and marker of oxygen radical injury [17]. Neutrophilic inflammation and oxidative stress are associated with altered lung development which can lead to BPD diagnosis, with preterm neonates also lacking anti-inflammatory and antioxidant defences [1, 2]. The presence of increased concentration of these markers in the exhaled breath of preterm-born infants aged 12 to 16 months cPNA suggests these inflammatory and oxidative stress processes continue beyond the first year of life. Neutrophil apoptosis is suppressed in the airways of preterm-born infants [19, 20], which may result in dysregulated inflammatory processes and contribute to chronic inflammation, possibly explaining the presence of neutrophilic inflammation into the first years of life.

The finding of elevated neutrophilic inflammation markers at 12–16 months in infants born very preterm is concerning. Elevated inflammation causes lung injury and alters development in the neonatal intensive care unit (NICU) period [21], and airway neutrophilia is associated with increased infection severity in diseases such as chronic obstructive pulmonary disease (COPD) [22]. The presence of elevated airway neutrophilic inflammation may increase the susceptibility of preterm-born children to more severe respiratory infections, which in turn may contribute to the higher rate of respiratory hospitalisations observed in the first years of life in this population [23]. Alveolar development continues throughout the early years of life [24] but may be disrupted when it occurs in the presence of inflammation and oxidative stress [25]. A recent study in mice found that neutrophilic inflammation during lung development prevents the normal assembly of elastin fibres around terminal airspaces leading to alveolar simplification and predisposing adult mice to COPD [26]. Increased neutrophilic inflammation and oxidative stress with normal or reduced eosinophilic inflammation is often observed in COPD, leading to protease imbalance and alveolar cell apoptosis [27]. These biomarkers may indicate a predisposition to COPD development in those born very preterm when considered in the context of evidence of structural lung damage on computed tomography and low and declining lung function observed during childhood in those born preterm [28–30]. Indeed, increased rates of COPD in those born preterm were reported recently [31]. Alternatively, elevated neutrophilia and oxidative stress markers may indicate a pathway to the development of neutrophilic asthma [32], particularly when considering the higher rates of asthma diagnosis in childhood in those born preterm compared to those born at term [33]. Additionally, recurrent infection, an altered immune response and airway microbiota play a role in neutrophilic/non-eosinophilic asthma, and these factors are similarly altered in those born preterm [34–36]. In the context of what is known about respiratory conditions like non-eosinophilic asthma and COPD, in addition to respiratory morbidity in preterm-born children, elevated markers of neutrophilic inflammation and oxidative stress may indicate the underlying disease process in this group. As such, clinical respiratory follow up of this population is warranted after discharge from the NICU, with the aim of identifying individuals at risk of persistent or progressive lung disease. Indeed, a recent European Respiratory Society guideline on the long-term management of children with BPD recommends monitoring this population with lung function [37]. This guideline also noted the urgent need for airway pathophysiological studies in this population in order to improve the evidence for clinical management guidelines for children with BPD [37]. As yet, there remains little co-ordinated respiratory follow-up for those surviving preterm birth.

Our study found no direct correlations of biomarker levels with gestational age, neonatal factors or respiratory symptom history, and no difference in biomarker levels between those with and without BPD. The absence of a direct association between EBC biomarkers at 12–16 months and indicators of early life respiratory morbidity may indicate that regardless of BPD diagnosis, preterm-born infants exhibit ongoing lung inflammation and oxidative stress. These findings are similar to those reported by Filippone et al. who found elevated 8-isoprostane in preterm-born adolescents both with and without BPD [7]. However, concentrations of 8-isoprostane in our study were lower than those reported by Filippone et al., which may be explained by differences in EBC collection devices [38]. Although reference ranges of 8-isoprostane in exhaled breath condensate from adults exist [38], none exist for leukotriene B4 or 8-isoprostane in exhaled breath condensate from infants, therefore a clinically relevant value cannot be defined. Additionally, EBC collection in infants occurs with nasal breathing rather than oral breathing as in older children and adults [8]. Moeller et al. found that oral EBC collection in infants yielded a greater sample volume than nasal EBC [39], however it is unknown if this effects biomarker concentration as well. The similarity of biomarker levels between those with and without BPD may also indicate a need for more objective, non-retrospective definition of BPD, such as those suggested more recently in several publications [40–42].

The lack of correlation with neonatal factors or symptoms may be explained by the inability to normalise EBC for variable dilution of airway surface liquid in water vapour, including by minute ventilation which previous studies have shown is associated with EBC volume in infants [39]. The variability in biomarker levels that we observed may suggest that aside from BPD diagnosis, any interruption of normal fetal lung development may result in chronic inflammation. Additionally, factors beyond the neonatal period, such as type, frequency and severity of respiratory infections in the first year of life are difficult to adjust for and may have a more pronounced or cumulative influence on persistent lung inflammation. Inflammation may be independent of BPD diagnosis or initial severity of lung disease. The parent-reported questionnaire only indicates the presence, rather than severity, of respiratory symptoms, where a more objective measure of respiratory morbidity would be more useful. Thes inability to normalise EBC markers may result in inexact quantification of biomarkers [43], which in turn may mask any associations with a more severe neonatal course or respiratory morbidity. Obtaining longitudinal lung health information from these infants would allow us to assess if exceeding a threshold concentration of these markers in EBC is associated with future respiratory morbidity. Additionally, further standardisation of EBC analysis will allow for more reliable biomarker quantification. Our study had limited sample numbers and larger studies would be more adequately powered to better establish an estimate of the true effect, define the clinically relevant differences and assess associations with potential predictors of inflammatory marker levels, including BPD status. Despite these limitations, increased inflammation and oxidative stress in the airways of infants born preterm regardless of BPD diagnosis may be an important contributor to the high rates of recurrent respiratory infection and hospitalisation in the first years observed in this population.

## Conclusions

Together, the results of the present study reinforce emerging evidence that neutrophilic inflammatory and oxidative stress processes persist in the airways of survivors of preterm birth regardless of BPD diagnosis. Although inflammatory biomarker levels were not directly associated with indicators of respiratory morbidity in the first year of life in this study, the elevation of these markers in survivors of preterm birth beyond 12 months of age may indicated increased risk of future respiratory morbidity as previously observed in the preterm-born population. Interventions that target neutrophilic inflammatory and oxidative stress processes may be useful in ameliorating the respiratory morbidity observed in survivors of preterm birth. The identification of elevated airway neutrophilic inflammation and oxidative stress at 9–16 months in those born preterm provides some insight into the pathogenesis of long-term respiratory morbidity in this population and identifies potential targets for intervention to preserve lung health in these children.

### Electronic supplementary material

Below is the link to the electronic supplementary material.

Supplementary Material 1

## Data Availability

Deidentified datasets generated during and/or analysed during the current study are available from the corresponding author on reasonable request to researchers who provide a methodologically sound proposal and have appropriate ethical and institutional approval.

## Abbreviations

BPD: bronchopulmonary dysplasia
EBC: exhaled breath condensate
cPNA: corrected postnatal age
ELISA: enzyme-linked immune-assays
NICU: neonatal intensive care unit
COPD: chronic obstructive pulmonary disease
CPAP: continuous positive airway pressure
