# Supplementary material for: Elevated leukotriene B4 and 8-isoprostane in exhaled breath condensate from preterm-born infants
Source: BMC Pediatr. 2023 Aug 5;23:386. doi: 10.1186/s12887-023-04210-y (PMC10403823; doi:10.1186/s12887-023-04210-y)
Supplement: Supplementary file 1 — Supplementary Material 1 [file 12887_2023_4210_MOESM1_ESM.pdf]

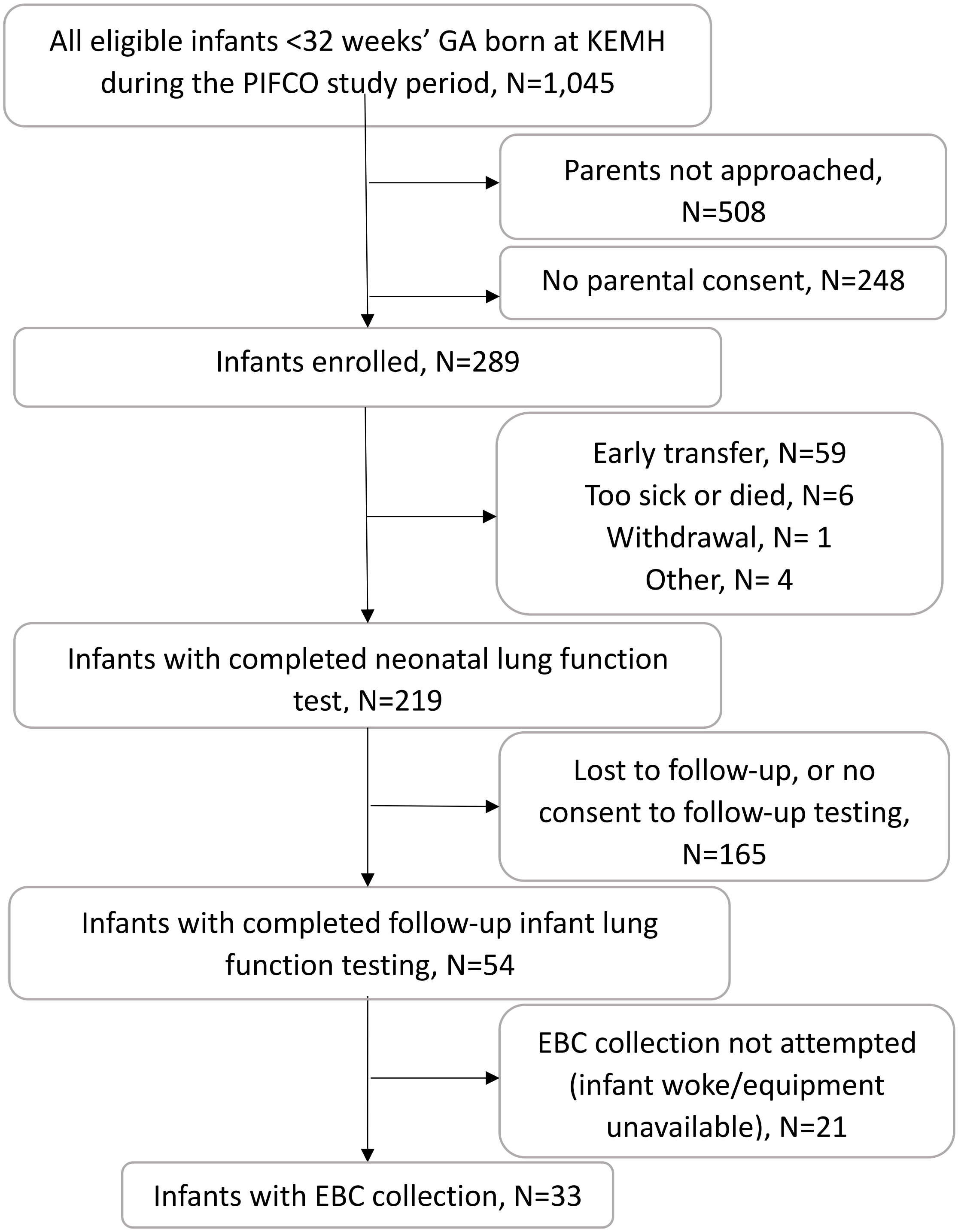

Supplementary Figure 1. Flowchart illustrating the recruitment process for participants in the current study as part of the larger Preterm Infant Functional and Clinical Outcomes (PIFCO) follow-up study.
